# 5-Fluoro-2-methyl-3-phenyl­sulfonyl-1-benzofuran

**DOI:** 10.1107/S1600536809055068

**Published:** 2010-01-09

**Authors:** Hong Dae Choi, Pil Ja Seo, Byeng Wha Son, Uk Lee

**Affiliations:** a Department of Chemistry, Dongeui University, San 24 Kaya-dong Busanjin-gu, Busan 614-714, Republic of Korea; b Department of Chemistry, Pukyong National University, 599-1 Daeyeon 3-dong, Nam-gu, Busan 608-737, Republic of Korea

## Abstract

There are two symmetry-independent mol­ecules, *A* and *B*, in the asymmetric unit of the title compound, C_15_H_11_FO_3_S. The crystal studied was an inversion twin with a 0.21 (12):0.79 (12) domain ratio. In the crystal structure, the two independent mol­ecules are related by a pseudo-inversion center. The dihedral angles formed by the phenyl ring and the plane of the benzofuran fragment are 80.2 (1)° in mol­ecule *A* and 80.7 (1)° in mol­ecule *B*. In the crystal structure, the *A* and *B* mol­ecules are linked by aromatic π–π inter­actions between the furan and benzene rings of neighbouring benzofuran systems; the centroid–centroid distances are 3.671 (7) and 3.715 (7) Å. In addition, the crystal structure also exhibits two weak non-classical inter­molecular C—H⋯O hydrogen bonds.

## Related literature

For the crystal structures of similar 5-halo-2-methyl-3-phenyl­sulfonyl-1-benzofuran derivatives, see: Choi *et al.* (2008*a* ▶,*b* ▶,*c* ▶). For natural products with benzofuran ring systems, see: Akgul & Anil (2003 ▶); Soekamto *et al.* (2003 ▶). For the biological activity of benzofuran compounds, see: Aslam *et al.* (2006 ▶); Galal *et al.* (2009 ▶).
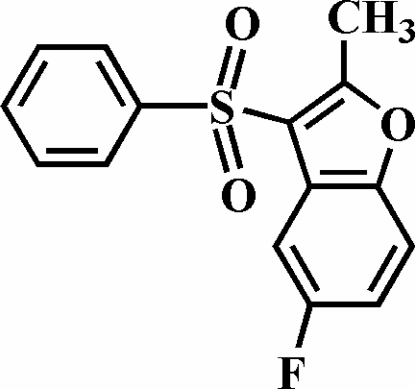


## Experimental

### 

#### Crystal data

C_15_H_11_FO_3_S
*M* *_r_* = 290.30
Monoclinic, 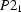

*a* = 7.377 (2) Å
*b* = 19.831 (4) Å
*c* = 9.025 (2) Å
β = 101.367 (3)°
*V* = 1294.4 (5) Å^3^
*Z* = 4
Mo *K*α radiation
μ = 0.27 mm^−1^
*T* = 173 K
0.40 × 0.20 × 0.05 mm

#### Data collection

Bruker SMART APEXII CCD diffractometer
Absorption correction: multi-scan (*SADABS*; Bruker, 2009 ▶) *T*_min_ = 0.930, *T*_max_ = 0.990
6055 measured reflections
3996 independent reflections
3154 reflections with *I* > 2σ(*I*)
*R*_int_ = 0.062

#### Refinement

*R*[*F* ^2^ > 2σ(*F* ^2^)] = 0.049
*wR*(*F* ^2^) = 0.115
*S* = 1.07
3996 reflections
362 parameters
1 restraint
H-atom parameters constrained
Δρ_max_ = 0.26 e Å^−3^
Δρ_min_ = −0.35 e Å^−3^
Absolute structure: Flack (1983 ▶), 1642 Friedel pairs
Flack parameter: 0.21 (12)

### 

Data collection: *APEX2* (Bruker, 2009 ▶); cell refinement: *SAINT* (Bruker, 2009 ▶); data reduction: *SAINT*; program(s) used to solve structure: *SHELXS97* (Sheldrick, 2008 ▶); program(s) used to refine structure: *SHELXL97* (Sheldrick, 2008 ▶); molecular graphics: *ORTEP-3* (Farrugia, 1997 ▶) and *DIAMOND* (Brandenburg, 1998 ▶); software used to prepare material for publication: *SHELXL97*.

## Supplementary Material

Crystal structure: contains datablocks global, I. DOI: 10.1107/S1600536809055068/ng2699sup1.cif

Structure factors: contains datablocks I. DOI: 10.1107/S1600536809055068/ng2699Isup2.hkl

Additional supplementary materials:  crystallographic information; 3D view; checkCIF report

## Figures and Tables

**Table 1:** Hydrogen-bond geometry (Å, °)

*D*—H⋯*A*	*D*—H	H⋯*A*	*D*⋯*A*	*D*—H⋯*A*
C3—H3⋯O6^i^	0.93	2.60	3.494 (6)	162
C26—H26⋯O2^ii^	0.93	2.55	3.479 (7)	174

Symmetry codes: (i) 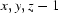; (ii) 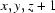.

**Fig. 1.**
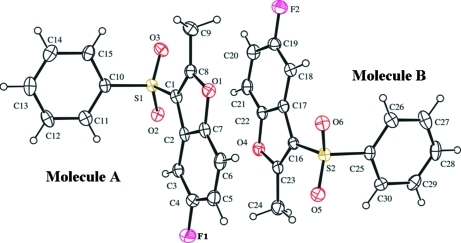
The molecular structure of the title compound with the atom numbering scheme. Displacement ellipsoids are drawn at the 50% probability level. H atoms are presented as a small spheres of arbitrary radius.

**Fig. 2.**
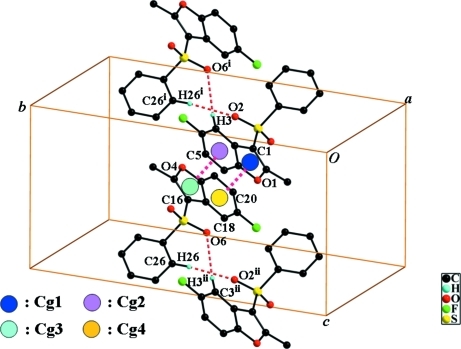
π–π and C—H···O interactions (dotted lines) in the crystal structure of the title compound. Cg denotes the ring centroid. [Symmetry codes: (i) x, y, z - 1; (ii) x, y, z + 1.]

## supplementary crystallographic information

### Comment

Molecules involving benzofuran skeleton have attracted considerable interest in the view of their presence in natural products (Akgul & Anil, 2003; Soekamto *et al.*, 2003) and their biological activity (Aslam *et al.*, 2006; Galal *et al.*, 2009). As a part of our continuing studies of the effect of side chain substituents on the solid state structures of 5-halo-2-methyl-3-phenylsulfonyl-1-benzofuran analogues (Choi *et al.*, 2008*a, b, c*), we report the crystal structure of the title compound (Fig. 1). The crystal studied was an inversion twin with a 0.21 (12):0.79 (12) domain ratio. It crystallized in the monoclinic space group P2_1_, with two symmetry-independent molecules, A and B, in the asymmetric unit.

The benzofuran unit is essentially planar, with a mean deviation of 0.011 (4) Å for A molecule and 0.007 (4) Å for B molecule, respectively, from the least-squares plane defined by the nine constituent atoms. In the title compound, the dihedral angles formed by the phenyl ring and the plane of the benzofuran fragment are 80.2 (1)° in molecule A and 80.7 (1)° in molecule B, respectively. In the crystal packing (Fig. 2), the A and B molecules are linked by two different aromatic π–π interactions; the first between the furan ring (Cg1) and an adjacent benzene ring (Cg4) [distance = 3.671 (7) Å], the second between the furan ring (Cg3) and an adjacent benzene ring (Cg2) [distance = 3.715 (7) Å], (Cg1, Cg2, Cg3, and Cg4 are the centroids of the C1/C2/C7/O1/C8 furan ring, the C2–C7 benzene ring, the C16/C17/C22/O4/C23 furan ring, and the C17–C22 benzene ring, respectively). The molecular packing (Fig. 2) is further stabilized by two non-classical intermolecular C—H···O hydrogen bonds; the first between the benzene H atom and the oxygen of the S═O unit, with a C3—H3···O6^i^, the second between the phenyl H atom and the oxygen of the S═O unit, with a C26—H26···O2^ii^, respectively (Table 1).

### Experimental

77% 3-Chloroperoxybenzoic acid (560 mg, 2.5 mmol) was added in small portions to a stirred solution of 5-fluoro-2-methyl-3-phenylsulfanyl-1-benzofuran (310 mg, 1.2 mmol) in dichloromethane (40 mL) at 273 K. After being stirred at room temperature for 5h, the mixture was washed with saturated sodium bicarbonate solution and the organic layer was separated, dried over magnesium sulfate, filtered and concentrated in vacuum. The residue was purified by column chromatography (chloroform) to afford the title compound as a colorless solid [yield 75%, m.p. 397–398 K; *R*_f_ = 0.55 (chloroform)]. Single crystals suitable for X-ray diffraction were prepared by slow evaporation of a solution of the title compound in benzene at room temperature.

### Refinement

All H atoms were positioned geometrically and refined using a riding model, with C—H = 0.93 Å for aromatic H atoms and 0.96 Å for methyl H atoms, and with *U*_iso_(H) = 1.2*U*_eq_(C) for aromatic H atoms and 1.5*U*_eq_(C) for methyl H atoms. The reported Flack parameter was obtained by TWIN/BASF procedure in SHELXL (Sheldrick, 2008).

### Crystal data

**Table:**

C_15_H_11_FO_3_S	*F*(000) = 600
*M**_r_* = 290.30	*D*_x_ = 1.490 Mg m^−^^3^
Monoclinic, *P*2_1_	Mo *K*α radiation, λ = 0.71073 Å
Hall symbol: p 2yb	Cell parameters from 2528 reflections
*a* = 7.377 (2) Å	θ = 2.3–25.7°
*b* = 19.831 (4) Å	µ = 0.27 mm^−^^1^
*c* = 9.025 (2) Å	*T* = 173 K
β = 101.367 (3)°	Block, colourless
*V* = 1294.4 (5) Å^3^	0.40 × 0.20 × 0.05 mm
*Z* = 4	

### Data collection

**Table:**

Bruker SMART APEXII CCD diffractometer	3996 independent reflections
Radiation source: Rotating Anode	3154 reflections with *I* > 2σ(*I*)
HELIOS	*R*_int_ = 0.062
Detector resolution: 10.0 pixels mm^-1^	θ_max_ = 25.0°, θ_min_ = 2.1°
φ and ω scans	*h* = −8→8
Absorption correction: multi-scan (*SADABS*; Bruker, 2009)	*k* = −19→23
*T*_min_ = 0.930, *T*_max_ = 0.990	*l* = −10→10
6055 measured reflections	

### Refinement

**Table:**

Refinement on *F*^2^	Secondary atom site location: difference Fourier map
Least-squares matrix: full	Hydrogen site location: difference Fourier map
*R*[*F*^2^ > 2σ(*F*^2^)] = 0.049	H-atom parameters constrained
*wR*(*F*^2^) = 0.115	*w* = 1/[σ^2^(*F*_o_^2^) + (0.0406*P*)^2^ + 0.9464*P*] where *P* = (*F*_o_^2^ + 2*F*_c_^2^)/3
*S* = 1.07	(Δ/σ)_max_ < 0.001
3996 reflections	Δρ_max_ = 0.26 e Å^−^^3^
362 parameters	Δρ_min_ = −0.35 e Å^−^^3^
1 restraint	Absolute structure: Flack (1983), 1642 Friedel pairs
Primary atom site location: structure-invariant direct methods	Flack parameter: 0.21 (12)

### Special details

**Table:**

Geometry. All esds (except the esd in the dihedral angle between two l.s. planes) are estimated using the full covariance matrix. The cell esds are taken into account individually in the estimation of esds in distances, angles and torsion angles; correlations between esds in cell parameters are only used when they are defined by crystal symmetry. An approximate (isotropic) treatment of cell esds is used for estimating esds involving l.s. planes.
Refinement. Refinement of F^2^ against ALL reflections. The weighted R-factor wR and goodness of fit S are based on F^2^, conventional R-factors R are based on F, with F set to zero for negative F^2^. The threshold expression of F^2^ > 2sigma(F^2^) is used only for calculating R-factors(gt) etc. and is not relevant to the choice of reflections for refinement. R-factors based on F^2^ are statistically about twice as large as those based on F, and R- factors based on ALL data will be even larger.

### Fractional atomic coordinates and isotropic or equivalent isotropic displacement parameters (Å^2^)

**Table:**

	*x*	*y*	*z*	*U*_iso_*/*U*_eq_	
S1	0.40869 (16)	0.34079 (7)	0.07406 (13)	0.0315 (3)	
S2	0.29228 (16)	0.56069 (6)	0.66799 (14)	0.0314 (3)	
O1	0.6428 (5)	0.40440 (18)	0.4794 (4)	0.0356 (9)	
O2	0.2917 (5)	0.38775 (19)	−0.0206 (4)	0.0366 (10)	
O3	0.3351 (5)	0.27849 (19)	0.1139 (4)	0.0419 (10)	
O4	0.0634 (5)	0.5048 (2)	0.2569 (4)	0.0380 (10)	
O5	0.3689 (5)	0.62390 (18)	0.6366 (4)	0.0396 (9)	
O6	0.4061 (5)	0.51239 (18)	0.7608 (4)	0.0370 (9)	
F1	0.5315 (5)	0.62712 (18)	0.1306 (4)	0.0595 (10)	
F2	0.1460 (5)	0.27594 (17)	0.5845 (4)	0.0573 (10)	
C1	0.5062 (6)	0.3838 (3)	0.2392 (5)	0.0292 (12)	
C2	0.5405 (6)	0.4548 (3)	0.2512 (5)	0.0277 (12)	
C3	0.5098 (7)	0.5105 (3)	0.1556 (6)	0.0326 (13)	
H3	0.4560	0.5063	0.0537	0.039*	
C4	0.5630 (8)	0.5711 (3)	0.2194 (6)	0.0409 (14)	
C5	0.6451 (8)	0.5809 (3)	0.3707 (7)	0.0438 (16)	
H5	0.6773	0.6240	0.4072	0.053*	
C6	0.6775 (7)	0.5265 (3)	0.4641 (7)	0.0388 (14)	
H6	0.7324	0.5311	0.5656	0.047*	
C7	0.6260 (7)	0.4647 (3)	0.4027 (6)	0.0323 (13)	
C8	0.5701 (7)	0.3559 (3)	0.3777 (6)	0.0321 (13)	
C9	0.5796 (8)	0.2868 (3)	0.4372 (6)	0.0403 (15)	
H9A	0.5129	0.2842	0.5183	0.048*	
H9B	0.7065	0.2747	0.4742	0.048*	
H9C	0.5257	0.2563	0.3582	0.048*	
C10	0.5988 (7)	0.3223 (3)	−0.0108 (6)	0.0301 (13)	
C11	0.6510 (7)	0.3687 (3)	−0.1085 (6)	0.0390 (14)	
H11	0.5833	0.4080	−0.1342	0.047*	
C12	0.8080 (7)	0.3549 (3)	−0.1675 (6)	0.0412 (15)	
H12	0.8470	0.3854	−0.2330	0.049*	
C13	0.9051 (8)	0.2966 (4)	−0.1293 (7)	0.0495 (17)	
H13	1.0108	0.2881	−0.1679	0.059*	
C14	0.8482 (8)	0.2507 (3)	−0.0353 (7)	0.0452 (15)	
H14	0.9139	0.2109	−0.0119	0.054*	
C15	0.6935 (7)	0.2634 (3)	0.0255 (6)	0.0394 (14)	
H15	0.6544	0.2323	0.0898	0.047*	
C16	0.1980 (6)	0.5209 (3)	0.4988 (5)	0.0275 (11)	
C17	0.1588 (7)	0.4501 (3)	0.4804 (6)	0.0300 (12)	
C18	0.1820 (7)	0.3933 (3)	0.5702 (6)	0.0341 (14)	
H18	0.2355	0.3952	0.6725	0.041*	
C19	0.1217 (7)	0.3339 (3)	0.5001 (7)	0.0366 (14)	
C20	0.0419 (8)	0.3273 (3)	0.3496 (7)	0.0431 (15)	
H20	0.0051	0.2852	0.3094	0.052*	
C21	0.0171 (7)	0.3837 (3)	0.2591 (6)	0.0409 (14)	
H21	−0.0357	0.3812	0.1568	0.049*	
C22	0.0738 (7)	0.4434 (3)	0.3272 (6)	0.0320 (13)	
C23	0.1387 (7)	0.5516 (3)	0.3642 (6)	0.0327 (13)	
C24	0.1323 (8)	0.6216 (3)	0.3081 (7)	0.0478 (16)	
H24A	0.0060	0.6345	0.2711	0.057*	
H24B	0.1999	0.6247	0.2278	0.057*	
H24C	0.1867	0.6511	0.3890	0.057*	
C25	0.0987 (7)	0.5777 (3)	0.7488 (6)	0.0293 (13)	
C26	0.0476 (7)	0.5332 (3)	0.8495 (6)	0.0375 (14)	
H26	0.1157	0.4941	0.8776	0.045*	
C27	−0.1081 (8)	0.5478 (3)	0.9085 (7)	0.0490 (17)	
H27	−0.1435	0.5187	0.9786	0.059*	
C28	−0.2091 (8)	0.6040 (3)	0.8651 (7)	0.0461 (17)	
H28	−0.3129	0.6131	0.9057	0.055*	
C29	−0.1597 (8)	0.6481 (3)	0.7610 (7)	0.0434 (15)	
H29	−0.2305	0.6863	0.7310	0.052*	
C30	−0.0042 (7)	0.6347 (3)	0.7022 (6)	0.0385 (13)	
H30	0.0307	0.6637	0.6319	0.046*	

### Atomic displacement parameters (Å^2^)

**Table:**

	*U*^11^	*U*^22^	*U*^33^	*U*^12^	*U*^13^	*U*^23^
S1	0.0254 (7)	0.0406 (8)	0.0272 (7)	−0.0029 (6)	0.0021 (5)	−0.0014 (6)
S2	0.0266 (7)	0.0380 (8)	0.0283 (7)	−0.0013 (6)	0.0019 (5)	−0.0018 (6)
O1	0.039 (2)	0.041 (3)	0.025 (2)	0.0029 (17)	0.0014 (16)	0.0001 (18)
O2	0.029 (2)	0.046 (3)	0.032 (2)	0.0034 (18)	−0.0032 (16)	−0.0049 (19)
O3	0.041 (2)	0.047 (3)	0.037 (2)	−0.0117 (18)	0.0056 (18)	0.0009 (19)
O4	0.040 (2)	0.047 (3)	0.027 (2)	0.0071 (18)	0.0053 (16)	0.0018 (19)
O5	0.035 (2)	0.039 (2)	0.045 (2)	−0.0052 (18)	0.0084 (17)	−0.0042 (19)
O6	0.030 (2)	0.046 (2)	0.033 (2)	0.0040 (17)	0.0001 (16)	0.0034 (19)
F1	0.083 (3)	0.043 (2)	0.057 (2)	0.0025 (19)	0.0235 (19)	0.0091 (18)
F2	0.083 (3)	0.043 (2)	0.047 (2)	−0.0026 (18)	0.0171 (19)	0.0020 (17)
C1	0.019 (3)	0.041 (4)	0.025 (3)	−0.002 (2)	0.001 (2)	−0.001 (2)
C2	0.020 (3)	0.043 (4)	0.020 (3)	−0.001 (2)	0.005 (2)	−0.004 (2)
C3	0.032 (3)	0.041 (4)	0.025 (3)	0.002 (2)	0.006 (2)	−0.001 (3)
C4	0.046 (3)	0.037 (4)	0.042 (3)	0.004 (3)	0.016 (3)	0.005 (3)
C5	0.041 (3)	0.046 (4)	0.047 (4)	−0.006 (3)	0.015 (3)	−0.012 (3)
C6	0.033 (3)	0.053 (4)	0.030 (3)	−0.004 (3)	0.006 (2)	−0.014 (3)
C7	0.026 (3)	0.046 (4)	0.025 (3)	0.002 (2)	0.005 (2)	−0.002 (3)
C8	0.026 (3)	0.038 (4)	0.032 (3)	0.004 (2)	0.006 (2)	−0.001 (3)
C9	0.043 (4)	0.047 (4)	0.028 (3)	−0.003 (3)	0.001 (3)	0.006 (3)
C10	0.030 (3)	0.043 (4)	0.016 (2)	−0.002 (2)	0.000 (2)	−0.004 (2)
C11	0.035 (3)	0.053 (4)	0.027 (3)	−0.004 (3)	−0.001 (2)	−0.002 (3)
C12	0.036 (3)	0.071 (5)	0.015 (3)	−0.009 (3)	0.002 (2)	−0.001 (3)
C13	0.038 (4)	0.074 (5)	0.037 (4)	0.004 (3)	0.011 (3)	−0.010 (3)
C14	0.042 (3)	0.054 (4)	0.036 (3)	0.012 (3)	−0.001 (3)	−0.010 (3)
C15	0.036 (3)	0.044 (4)	0.038 (3)	0.002 (3)	0.006 (3)	−0.011 (3)
C16	0.025 (3)	0.034 (3)	0.024 (3)	0.003 (2)	0.004 (2)	0.003 (2)
C17	0.019 (3)	0.040 (4)	0.031 (3)	0.003 (2)	0.004 (2)	0.001 (2)
C18	0.033 (3)	0.041 (4)	0.030 (3)	−0.001 (2)	0.008 (2)	0.000 (3)
C19	0.033 (3)	0.035 (4)	0.042 (3)	0.000 (3)	0.010 (3)	0.005 (3)
C20	0.038 (3)	0.050 (4)	0.040 (3)	−0.009 (3)	0.006 (3)	−0.012 (3)
C21	0.039 (3)	0.057 (4)	0.026 (3)	0.000 (3)	0.005 (2)	−0.011 (3)
C22	0.027 (3)	0.045 (4)	0.024 (3)	0.002 (2)	0.005 (2)	−0.001 (3)
C23	0.030 (3)	0.039 (4)	0.031 (3)	0.001 (2)	0.012 (2)	0.000 (3)
C24	0.054 (4)	0.051 (4)	0.040 (4)	0.015 (3)	0.013 (3)	0.007 (3)
C25	0.023 (3)	0.039 (4)	0.024 (3)	−0.002 (2)	0.001 (2)	−0.007 (3)
C26	0.027 (3)	0.054 (4)	0.029 (3)	−0.003 (2)	−0.001 (2)	0.003 (3)
C27	0.036 (3)	0.081 (5)	0.031 (3)	−0.004 (3)	0.008 (3)	0.004 (3)
C28	0.031 (3)	0.077 (5)	0.029 (3)	−0.004 (3)	0.002 (3)	−0.023 (3)
C29	0.035 (3)	0.043 (4)	0.050 (4)	0.009 (3)	0.003 (3)	−0.011 (3)
C30	0.043 (3)	0.034 (3)	0.041 (3)	0.000 (3)	0.013 (3)	−0.001 (3)

### Geometric parameters (Å, °)

**Table:**

S1—O3	1.424 (4)	C12—C13	1.368 (8)
S1—O2	1.432 (4)	C12—H12	0.9300
S1—C1	1.744 (5)	C13—C14	1.365 (8)
S1—C10	1.764 (6)	C13—H13	0.9300
S2—O5	1.426 (4)	C14—C15	1.383 (8)
S2—O6	1.430 (4)	C14—H14	0.9300
S2—C16	1.738 (5)	C15—H15	0.9300
S2—C25	1.759 (5)	C16—C23	1.352 (7)
O1—C8	1.364 (6)	C16—C17	1.437 (7)
O1—C7	1.375 (6)	C17—C18	1.378 (7)
O4—C22	1.369 (6)	C17—C22	1.408 (7)
O4—C23	1.377 (6)	C18—C19	1.369 (8)
F1—C4	1.363 (6)	C18—H18	0.9300
F2—C19	1.371 (7)	C19—C20	1.377 (8)
C1—C8	1.364 (7)	C20—C21	1.375 (8)
C1—C2	1.431 (7)	C20—H20	0.9300
C2—C3	1.392 (7)	C21—C22	1.361 (8)
C2—C7	1.402 (7)	C21—H21	0.9300
C3—C4	1.357 (8)	C23—C24	1.474 (7)
C3—H3	0.9300	C24—H24A	0.9600
C4—C5	1.393 (8)	C24—H24B	0.9600
C5—C6	1.361 (8)	C24—H24C	0.9600
C5—H5	0.9300	C25—C26	1.372 (7)
C6—C7	1.368 (8)	C25—C30	1.381 (7)
C6—H6	0.9300	C26—C27	1.389 (8)
C8—C9	1.469 (8)	C26—H26	0.9300
C9—H9A	0.9600	C27—C28	1.356 (8)
C9—H9B	0.9600	C27—H27	0.9300
C9—H9C	0.9600	C28—C29	1.383 (8)
C10—C15	1.368 (7)	C28—H28	0.9300
C10—C11	1.380 (8)	C29—C30	1.381 (8)
C11—C12	1.394 (8)	C29—H29	0.9300
C11—H11	0.9300	C30—H30	0.9300
			
O3—S1—O2	120.0 (2)	C12—C13—H13	119.7
O3—S1—C1	108.8 (2)	C13—C14—C15	120.3 (6)
O2—S1—C1	106.9 (2)	C13—C14—H14	119.8
O3—S1—C10	107.7 (2)	C15—C14—H14	119.8
O2—S1—C10	108.3 (2)	C10—C15—C14	118.8 (6)
C1—S1—C10	104.1 (2)	C10—C15—H15	120.6
O5—S2—O6	119.7 (2)	C14—C15—H15	120.6
O5—S2—C16	109.3 (2)	C23—C16—C17	108.3 (4)
O6—S2—C16	107.3 (2)	C23—C16—S2	126.0 (4)
O5—S2—C25	107.5 (2)	C17—C16—S2	125.6 (4)
O6—S2—C25	108.4 (2)	C18—C17—C22	118.7 (5)
C16—S2—C25	103.5 (2)	C18—C17—C16	137.1 (5)
C8—O1—C7	106.9 (4)	C22—C17—C16	104.3 (4)
C22—O4—C23	107.2 (4)	C19—C18—C17	116.1 (5)
C8—C1—C2	107.8 (4)	C19—C18—H18	122.0
C8—C1—S1	126.5 (4)	C17—C18—H18	122.0
C2—C1—S1	125.7 (4)	C18—C19—F2	118.0 (5)
C3—C2—C7	118.7 (5)	C18—C19—C20	125.1 (6)
C3—C2—C1	136.7 (5)	F2—C19—C20	116.9 (5)
C7—C2—C1	104.5 (4)	C21—C20—C19	119.3 (6)
C4—C3—C2	116.2 (5)	C21—C20—H20	120.3
C4—C3—H3	121.9	C19—C20—H20	120.3
C2—C3—H3	121.9	C22—C21—C20	116.4 (5)
C3—C4—F1	118.1 (5)	C22—C21—H21	121.8
C3—C4—C5	125.0 (6)	C20—C21—H21	121.8
F1—C4—C5	116.9 (6)	C21—C22—O4	125.5 (5)
C6—C5—C4	119.1 (6)	C21—C22—C17	124.4 (5)
C6—C5—H5	120.5	O4—C22—C17	110.1 (5)
C4—C5—H5	120.5	C16—C23—O4	110.0 (4)
C5—C6—C7	117.2 (5)	C16—C23—C24	135.6 (5)
C5—C6—H6	121.4	O4—C23—C24	114.3 (5)
C7—C6—H6	121.4	C23—C24—H24A	109.5
C6—C7—O1	125.8 (5)	C23—C24—H24B	109.5
C6—C7—C2	123.8 (5)	H24A—C24—H24B	109.5
O1—C7—C2	110.4 (5)	C23—C24—H24C	109.5
C1—C8—O1	110.5 (5)	H24A—C24—H24C	109.5
C1—C8—C9	134.2 (5)	H24B—C24—H24C	109.5
O1—C8—C9	115.3 (4)	C26—C25—C30	121.5 (5)
C8—C9—H9A	109.5	C26—C25—S2	120.2 (4)
C8—C9—H9B	109.5	C30—C25—S2	118.2 (4)
H9A—C9—H9B	109.5	C25—C26—C27	118.4 (5)
C8—C9—H9C	109.5	C25—C26—H26	120.8
H9A—C9—H9C	109.5	C27—C26—H26	120.8
H9B—C9—H9C	109.5	C28—C27—C26	120.6 (6)
C15—C10—C11	121.9 (5)	C28—C27—H27	119.7
C15—C10—S1	119.1 (4)	C26—C27—H27	119.7
C11—C10—S1	119.0 (4)	C27—C28—C29	120.9 (6)
C10—C11—C12	118.1 (6)	C27—C28—H28	119.6
C10—C11—H11	120.9	C29—C28—H28	119.6
C12—C11—H11	120.9	C30—C29—C28	119.3 (6)
C13—C12—C11	120.1 (6)	C30—C29—H29	120.4
C13—C12—H12	119.9	C28—C29—H29	120.4
C11—C12—H12	119.9	C25—C30—C29	119.3 (5)
C14—C13—C12	120.7 (6)	C25—C30—H30	120.4
C14—C13—H13	119.7	C29—C30—H30	120.4
			
O3—S1—C1—C8	24.1 (5)	O5—S2—C16—C23	−24.0 (5)
O2—S1—C1—C8	155.0 (4)	O6—S2—C16—C23	−155.2 (4)
C10—S1—C1—C8	−90.6 (5)	C25—S2—C16—C23	90.3 (5)
O3—S1—C1—C2	−158.9 (4)	O5—S2—C16—C17	160.2 (4)
O2—S1—C1—C2	−28.0 (5)	O6—S2—C16—C17	29.1 (5)
C10—S1—C1—C2	86.5 (5)	C25—S2—C16—C17	−85.5 (4)
C8—C1—C2—C3	−179.6 (6)	C23—C16—C17—C18	−179.4 (6)
S1—C1—C2—C3	2.8 (8)	S2—C16—C17—C18	−3.0 (8)
C8—C1—C2—C7	−0.1 (5)	C23—C16—C17—C22	−0.1 (5)
S1—C1—C2—C7	−177.6 (4)	S2—C16—C17—C22	176.3 (4)
C7—C2—C3—C4	−1.4 (7)	C22—C17—C18—C19	1.1 (7)
C1—C2—C3—C4	178.1 (5)	C16—C17—C18—C19	−179.7 (5)
C2—C3—C4—F1	−178.5 (4)	C17—C18—C19—F2	178.5 (4)
C2—C3—C4—C5	0.3 (8)	C17—C18—C19—C20	0.2 (8)
C3—C4—C5—C6	0.5 (9)	C18—C19—C20—C21	−0.5 (9)
F1—C4—C5—C6	179.3 (5)	F2—C19—C20—C21	−178.9 (5)
C4—C5—C6—C7	−0.2 (8)	C19—C20—C21—C22	−0.4 (8)
C5—C6—C7—O1	−178.2 (5)	C20—C21—C22—O4	179.2 (5)
C5—C6—C7—C2	−0.9 (8)	C20—C21—C22—C17	1.7 (8)
C8—O1—C7—C6	178.1 (5)	C23—O4—C22—C21	−178.6 (5)
C8—O1—C7—C2	0.5 (5)	C23—O4—C22—C17	−0.9 (5)
C3—C2—C7—C6	1.8 (7)	C18—C17—C22—C21	−2.1 (8)
C1—C2—C7—C6	−177.9 (5)	C16—C17—C22—C21	178.4 (5)
C3—C2—C7—O1	179.4 (4)	C18—C17—C22—O4	−179.9 (4)
C1—C2—C7—O1	−0.3 (5)	C16—C17—C22—O4	0.6 (5)
C2—C1—C8—O1	0.4 (5)	C17—C16—C23—O4	−0.5 (5)
S1—C1—C8—O1	177.9 (3)	S2—C16—C23—O4	−176.8 (3)
C2—C1—C8—C9	−178.3 (5)	C17—C16—C23—C24	177.9 (5)
S1—C1—C8—C9	−0.8 (8)	S2—C16—C23—C24	1.5 (9)
C7—O1—C8—C1	−0.6 (5)	C22—O4—C23—C16	0.9 (5)
C7—O1—C8—C9	178.4 (4)	C22—O4—C23—C24	−177.9 (4)
O3—S1—C10—C15	−26.1 (5)	O5—S2—C25—C26	−150.5 (4)
O2—S1—C10—C15	−157.2 (4)	O6—S2—C25—C26	−19.8 (5)
C1—S1—C10—C15	89.3 (5)	C16—S2—C25—C26	93.9 (4)
O3—S1—C10—C11	155.8 (4)	O5—S2—C25—C30	32.8 (5)
O2—S1—C10—C11	24.6 (5)	O6—S2—C25—C30	163.5 (4)
C1—S1—C10—C11	−88.8 (4)	C16—S2—C25—C30	−82.8 (5)
C15—C10—C11—C12	−1.8 (8)	C30—C25—C26—C27	−2.2 (8)
S1—C10—C11—C12	176.3 (4)	S2—C25—C26—C27	−178.8 (4)
C10—C11—C12—C13	0.5 (8)	C25—C26—C27—C28	1.3 (8)
C11—C12—C13—C14	1.0 (9)	C26—C27—C28—C29	0.1 (9)
C12—C13—C14—C15	−1.4 (9)	C27—C28—C29—C30	−0.7 (9)
C11—C10—C15—C14	1.4 (8)	C26—C25—C30—C29	1.6 (8)
S1—C10—C15—C14	−176.7 (4)	S2—C25—C30—C29	178.3 (4)
C13—C14—C15—C10	0.2 (8)	C28—C29—C30—C25	−0.1 (9)

### Hydrogen-bond geometry (Å, °)

**Table:**

*D*—H···*A*	*D*—H	H···*A*	*D*···*A*	*D*—H···*A*
C3—H3···O6^i^	0.93	2.60	3.494 (6)	162
C26—H26···O2^ii^	0.93	2.55	3.479 (7)	174

Symmetry codes: (i) *x*, *y*, *z*−1; (ii) *x*, *y*, *z*+1.

## References

[bb1] Akgul, Y. Y. & Anil, H. (2003). *Phytochemistry*, **63**, 939–943. 10.1016/s0031-9422(03)00357-1 12895543

[bb2] Aslam, S. N., Stevenson, P. C., Phythian, S. J., Veitch, N. C. & Hall, D. R. (2006). *Tetrahedron*, **62**, 4214–4226.

[bb3] Brandenburg, K. (1998). *DIAMOND* Crystal Impact GbR, Bonn, Germany.

[bb4] Bruker (2009). *SADABS*, *APEX2* and *SAINT* Bruker AXS Inc., Madison, Wisconsin, USA.

[bb5] Choi, H. D., Seo, P. J., Son, B. W. & Lee, U. (2008*a*). *Acta Cryst.* E**64**, o793. 10.1107/S1600536808008489 PMC2961177 21202285

[bb6] Choi, H. D., Seo, P. J., Son, B. W. & Lee, U. (2008*b*). *Acta Cryst.* E**64**, o930. 10.1107/S1600536808011240 PMC2961136 21202411

[bb7] Choi, H. D., Seo, P. J., Son, B. W. & Lee, U. (2008*c*). *Acta Cryst.* E**64**, o1190. 10.1107/S1600536808015699 PMC2961728 21202832

[bb8] Farrugia, L. J. (1997). *J. Appl. Cryst.* **30**, 565.

[bb9] Flack, H. D. (1983). *Acta Cryst.* A**39**, 876–881.

[bb10] Galal, S. A., Abd El-All, A. S., Abdallah, M. M. & El-Diwani, H. I. (2009). *Bioorg. Med. Chem. Lett* **19**, 2420–2428. 10.1016/j.bmcl.2009.03.069 19345581

[bb11] Sheldrick, G. M. (2008). *Acta Cryst.* A**64**, 112–122. 10.1107/S0108767307043930 18156677

[bb12] Soekamto, N. H., Achmad, S. A., Ghisalberti, E. L., Hakim, E. H. & Syah, Y. M. (2003). *Phytochemistry*, **64**, 831–834. 10.1016/j.phytochem.2003.08.009 14559276

